# Circulating CD62E^+^ Microparticles and Cardiovascular Outcomes

**DOI:** 10.1371/journal.pone.0035713

**Published:** 2012-04-26

**Authors:** Soon-Tae Lee, Kon Chu, Keun-Hwa Jung, Jeong-Min Kim, Hye-Jin Moon, Jae-Jun Bahn, Woo-Seok Im, Junsang Sunwoo, Jangsup Moon, Manho Kim, Sang Kun Lee, Jae-Kyu Roh

**Affiliations:** 1 Department of Neurology, Biomedical Research Institute, Seoul National University Hospital, Seoul, South Korea; 2 Program in Neuroscience, Neuroscience Research Institute of Seoul National University Medical Research Center, Seoul National University College of Medicine, Seoul, South Korea; University of Queensland, Australia

## Abstract

**Background:**

Activated endothelial cells release plasma membrane submicron vesicles expressing CD62E (E-selectin) into blood, known as endothelial microparticles (EMPs). We studied whether the levels of endothelial microparticles expressing CD62E^+^, CD31^+^/Annexin-V^+^, or CD31^+^/CD42^−^ predict cardiovascular outcomes in patients with stroke history.

**Methods/Principal Findings:**

Patients with stroke history at least 3 months prior to enrolment were recruited. Peripheral blood EMP levels were measured by flow cytometry. Major cardiovascular events and death were monitored for 36 months. Three hundred patients were enrolled, of which 298 completed the study according to protocol. Major cardiovascular events occurred in 29 patients (9.7%). Nine patients died, five from cardiovascular causes. Cumulative event-free survival rates were lower in patients with high levels of CD62E^+^ microparticles. Multivariate Cox regression analysis adjusted for cardiovascular risk factors, medications and stroke etiologic groups showed an association between a high CD62E^+^ microparticle level and a risk of major cardiovascular events and hospitalization. Levels of other kinds of EMPs expressing CD31^+^/Annexin-V^+^ or CD31^+^/CD42^−^ markers were not predictive of cardiovascular outcomes.

**Conclusion:**

A high level of CD62E^+^ microparticles is associated with cardiovascular events in patients with stroke history, suggesting that the systemic endothelial activation increases the risk for cardiovascular morbidities.

## Introduction

Endothelial activation or damage plays a pivotal role in atherosclerosis [1], [2]. Apoptotic and activated endothelial cells release submicron vesicles called endothelial microparticles (EMPs) from the plasma membrane. The level of these vesicles is a sensitive indicator of the nature and extent of endothelial injury and activation in coronary or peripheral artery diseases [3], [4], [5], [6], [7]. Elevated levels of EMPs, mostly defined as CD31^+^/Annexin-V^+^ or CD31^+^/CD42^−^ microparticles, have been found in various vascular disorders [8]–[13]. Moreover, EMPs accumulate in atherosclerotic lesions and influence propagation of atherosclerosis [14].

However, the role of EMPs in the prognosis of cardiovascular diseases remains to be determined. Several different markers are used to identify EMPs, and each marker has different clinical implications. For example, apoptotic endothelial cells shed EMPs in which constitutive markers such as CD31^+^/Annexin-V^+^ predominate, whereas activated endothelial cells shed EMPs in which inducible markers such as CD62E (E-selectin) predominate [4]. Thus, when endothelial cells were activated by tumor necrosis factor-α to express surface adhesion molecules which participate in leukocyte and platelet recruitments, the activated endothelial cells release CD62E^+^ microparticles [4]. Meanwhile, apoptotic endothelial cells induced by growth factor deprivation release Annexin-V^+^ expressing microparticles [4]. In stroke patients, higher level of circulating CD31^+^/Annexin-V^+^ microparticles is associated with increased risk of intracranial stenosis, whereas higher level of CD62E^+^ microparticles is associated with extracranial carotid atherosclerosis [8]. In pulmonary hypertension, an elevated level of only CD62E^+^ microparticles is predictive of a poor outcome [15]. Finally, in obstructive sleep apnea, continuous positive airway pressure therapy reduces the level of CD62E^+^ but not CD31^+^/CD42^−^ microparticles [16].

Given this evidence, we hypothesized that the higher level of CD62E^+^ microparticles could be useful for predicting the poor cardiovascular prognosis of non-coronary artery diseases. We also hypothesized that microparticles expressing CD31^+^/Annexin-V^+^ or CD31^+^/CD42^−^ might have limited but still meaningful predictive values for the poor cardiovascular prognosis as well. We therefore performed a prospective study in which we examined the association between the level of endothelial microparticles and cardiovascular outcomes in patients with old stroke.

## Methods

### Subject enrollment

This study was approved by the institutional review board of Seoul National University Hospital, and all subjects provided written informed consent prior to enrollment. Patients who experienced a first-ever stroke at least 3 months prior to enrolment were recruited at the Stroke Clinic of the Department of Neurology, Seoul National University Hospital. Because several conditions are known to increase the level of EMPs, subjects were excluded if they had one of the following: symptomatic coronary artery disease, symptomatic peripheral artery disease, or stroke of other determined etiology such as nonatherosclerotic vasculopathy and hypercoagulable states. We have previously reported that patients with recent stroke have high levels of EMPs [8], and those with recent stroke within the 3 months before enrolment were excluded in the current study.

### Subject assessments

At enrolment, citrated whole blood (5 ml), demographic data, and medical history were collected from each patient. The total cardiovascular risk factor burden at baseline was calculated for each patient using Framingham risk factor scores [17]. Stroke etiologies at baseline were determined according to the TOAST classification [18]. All patients were followed-up every 3 to 6 months for 36 months to collect information about the occurrence of the following outcomes: first major cardiovascular event, including acute myocardial infarction or new coronary artery disease, stroke, hospitalization due to cardiovascular events, revascularization procedure, and death from cardiovascular causes; acute myocardial infarction and new coronary artery disease; revascularization in coronary, peripheral, and cerebral arteries; hospitalization due to cardiovascular events; ischemic or hemorrhagic strokes; death from cardiovascular causes; and death from any cause.

### Measurement of EMP levels

EMPs were quantified as described previously [19]. Briefly, citrated whole blood was centrifuged at 1500× g for 15 min to prepare platelet-rich plasma. The platelet-rich plasma was further centrifuged for 2 min at 13,000× g to obtain platelet-poor plasma, which was stored at −70°C. The platelet-poor plasma was thawed, and a 50-µL aliquot was mixed on an orbital shaker (120 rpm) for 30 min at room temperature with phycoerythrin-labeled anti-CD62E antibody (2 µL; BD Biosciences, San Jose, CA) or with a mixture of phycoerythrin-labeled anti-CD31, fluorescein isothiocynate-labeled anti-CD42b, and allophycocyanin-labeled Annexin-V antibody (2 µL each; BD Biosciences). The samples were then diluted to 1 ml with phosphate-buffered saline and analyzed with a FACS II flow cytometer (BD Biosciences) at the medium flow-rate setting and a 30-second stop time. EMPs were primarily defined as particles <1 µm expressing CD62E. Microbeads from a FACS Size Calibration Kit (Invitrogen, Carlsbad, CA) were used for size calibration. Apoptotic EMPs we defined as the sum of CD31^+^Annexin V^+^ and CD31^+^/CD42^−^ EMPs. Values are reported as counts/µL of platelet-poor plasma.

### Calculations and statistical analyses

The patients were stratified into high and low groups according to their levels of CD62E^+^ microparticles, with each group including 50% of the patients. The assumption of a normal distribution was determined by Kolmogorov-Smirnov test. Differences between continuous values with a normal distribution were analyzed by Student's *t*-test. Differences between values with non-normal distributions were analyzed by Mann-Whitney U test. Differences between categorical variables were analyzed by chi-square test. Univariate correlations between clinical factors and EMP groups were assessed using Pearson's correlation coefficients for continuous values and Spearman's correlation coefficients for categorical values. Baseline demographic characteristics, stroke etiologic groups, and medication use were analyzed using a multiple logistic regression model to identify independent predictors of cardiovascular outcomes. A multivariate Cox's proportional-hazards regression analysis was performed to determine the association between EMP counts and each outcome. Analyses were adjusted for advanced age (≥65 years); sex; hypertension; diabetes; dyslipidemia; smoking; high cardioembolic risk; coronary artery disease; treatment with antiplatelet agents, anticoagulation, angiotensin converting enzyme inhibitors/angiotensin II receptor blockers, beta-blockers, calcium-channel blocker, diuretics, or statins; and stroke etiologic groups. Survival was determined by the Kaplan-Meier method and the log-rank test was used to determine the statistical significance of differences in event-free survival. Data are expressed as means ± standard deviations or as a number (percentages). SPSS software (SPSS 17.0, SPSS Inc., Chicago, IL) was used for analyses, and a two-tailed probability value less than 0.05 was considered to be significant. All investigators performing event classifications were blinded to patients' EMP levels.

## Results

### Baseline characteristics

Three hundred patients were enrolled. Two patients were lost to follow-up before 36 months, so that 298 patients completed the study according to protocol. The patient characteristics are summarized in Table S1. At enrollment, the number of CD62E^+^ microparticles/µL ranged from 0 to 1223, with a mean of 87±128 (after common logarithmic transformation, 1.74±0.38). Because levels of EMPs were not normally distributed according to a Kolmogorov–Smirnov test (P<0.001), the patients were stratified into two equal groups according to their levels of CD62E^+^ microparticles: counts/µL ranged from 0 to 53 for the low CD62E^+^ microparticle group and from 54 to 1223 for the high CD62E^+^ microparticle group. According to univariate analysis, none of the baseline characteristics, risk factors, medication profiles, and the mechanisms of previous stroke were significantly associated with the level of CD62E^+^ microparticles at enrollment (Table S1). In addition, the level of CD62E^+^ microparticle was not correlated with the time from stroke onset (Figure S1).

### Cardiovascular outcomes

At 36 months, nine patients (3.0%) had died, including five from cardiovascular causes (1.7%), one from pneumonia, one from renal insufficiency, one from prostate cancer, and one for an unclassified reason (Table 1). Twenty-nine patients (9.7%) had major cardiovascular events, including 11 (3.7%) that had an acute myocardial infarction or new coronary artery disease, five (1.7%) that required revascularization, and 23 (7.7%) that were admitted to a hospital due to cardiovascular events. The incidences of a first major cardiovascular event and of hospitalization were higher in the high CD62E^+^ microparticle group than in the low CD62E^+^ microparticle group (P = 0.011 and 0.017, respectively) (Table 1).

**Table 1:** Frequency of cardiovascular events, hospitalization due to cardiovascular events, and death 36 months after enrollment.*

Events	Total (*N* = 298)	Low CD62E^+^ microparticle group (*N* = 149)	High CD62E^+^ microparticle group (*N* = 149)	P-value†
All major cardiovascular events	29 (9.7)	8 (5.4)	21 (14.1)	0.011
Myocardial infarction or new CAD	11 (3.7)	3 (2.0)	8 (5.4)	0.124
Revascularization	5 (1.7)	1 (0.7)	4 (2.7)	0.176
Hospitalization‡	23 (7.7)	6 (4.0)	17 (11.4)	0.017
All stroke	17 (5.6)	6 (4.0)	11 (7.4)	0.212
Ischemic stroke	13 (4.4)	4 (2.7)	9 (6.0)	0.156
Hemorrhagic stroke	4 (1.3)	2 (1.3)	2 (1.3)	1.000
Death from cardiovascular cause	5 (1.7)	1 (0.7)	4 (2.7)	0.176
Death from any cause	9 (3.0)	3 (2.0)	6 (4.0)	0.310

* Values are numbers of patients in each category, with the percent of the total in the group in brackets. CAD denotes coronary artery disease.

† *P* values were determined by chi-square test.

‡ Hospitalization was due to cardiovascular events, including recurrent angina, congestive heart failure, myocardial infarction, and stroke.

### Association between cardiovascular outcomes and patient characteristics

Univariate analysis indicated an association between all major cardiovascular events and advanced age (P = 0.007), known coronary artery disease (P = 0.009), and a high CD62E^+^ microparticle level (P = 0.017). The incidence of myocardial infarction and new coronary artery disease was associated with known coronary artery disease (P = 0.002). The incidence of hospitalization was associated with advanced age (P = 0.009), known coronary artery disease (P = 0.014), and a high level of CD62E^+^ microparticles (P = 0.025). The incidence of stroke was associated with the use of anticoagulants (P = 0.034) and a lower incidence of stroke with the use of calcium-channel blocker (P = 0.044). The incidence of ischemic stroke was significantly associated with the use anticoagulants (P = 0.047). Other cardiovascular outcomes, including the incidences of revascularization, hemorrhagic stroke, death from cardiovascular causes, and death from any causes were not significantly associated with demographic factors or the CD62E^+^ microparticle level (data not shown). Also, outcomes were not significantly associated with any of the stroke etiologic groups (data not shown).

Multivariate regression analysis found that the occurrence of major cardiovascular events was associated with a high level of CD62E^+^ microparticles (HR, 3.73; 95% CI, 1.39 to 10.07; P = 0.009), advanced age (HR, 3.36; 95% CI, 1.25 to 10.36; P = 0.018), coronary artery disease (HR, 4.70; 95% CI, 1.06 to 20.92; P = 0.042), and the use of anticoagulants (HR, 10.23; 95% CI, 1.56 to 66.92; P = 0.015). The occurrence of hospitalization was significantly associated with a high CD62E^+^ microparticle level (HR, 4.00; 95% CI, 1.25 to 12.48; P = 0.019), advanced age (HR, 4.44; 95% CI, 1.27 to 15.49; P = 0.019), coronary artery disease (HR, 5.13; 95% CI, 1.04 to 25.25; P = 0.044), and the use of anticoagulants (HR, 19.32; 95% CI, 2.60 to 143.50; P = 0.004), and a lack of hospitalization was significantly associated with the use of calcium-channel blockers (HR, 0.28; 95% CI, 0.08 to 0.94; P = 0.039). The incidence of all stroke was significantly associated with advanced age (HR, 4.07; 95% CI, 1.01 to 16.38; P = 0.048) and the use of anticoagulants (HR, 21.03; 95% CI, 2.45 to 180.64; P = 0.006), and a lack of stroke was associated with the use of calcium-channel blockers (HR, 0.153; 95% CI, 0.03 to 0.77; P = 0.023). The incidence of ischemic stroke was significantly associated with of the use of anticoagulants (HR, 13.14; 95% CI, 1.31 to 132.04; P = 0.029). Other cardiovascular outcomes, including the incidences of revascularization, hemorrhagic stroke, death from cardiovascular causes, and death from any causes were not significantly associated with any demographic factor, stroke etiology group, or CD62E^+^ microparticle level (data not shown).

### High CD62E^+^ microparticle and increased risks for all major cardiovascular events and hospitalization

**Figure 1:**
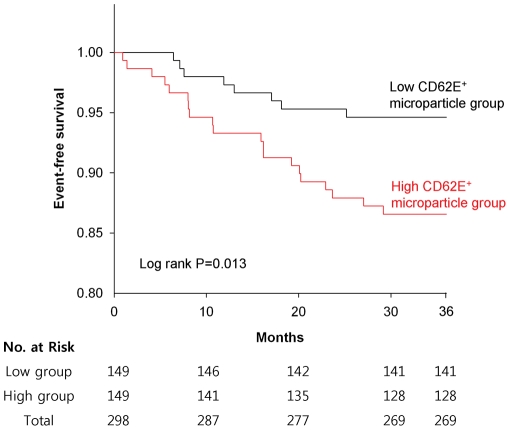
Cumulative event-free survival according to all cardiovascular events stratified by the level (low, high) of circulating CD62E^+^ microparticles at enrollment.

**Figure 2:**
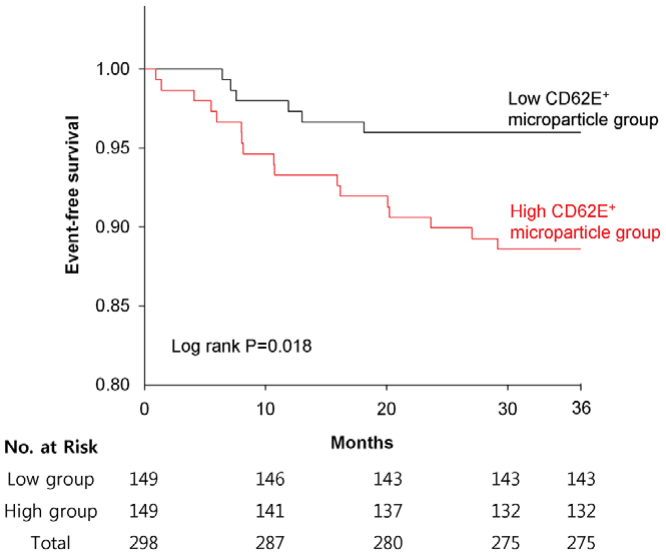
Cumulative event-free survival according to hospitalization stratified by the level (low, high) of circulating CD62E^+^ microparticles at enrollment.

Cumulative event-free survival rates were higher in the low CD62E^+^ microparticle group for all major cardiovascular events (P = 0.013; Fig. 1) and hospitalization (P = 0.018; Fig. 2). In addition, univariate Cox regression analysis found that a high level of CD62E^+^ microparticles was significantly associated with an increased risk for all major cardiovascular events and for hospitalization due to cardiovascular events (Table 2). Multivariate Cox regression analysis adjusting for advanced age, sex, cardiovascular risk factors, concomitant drug therapies, and stroke etiology groups, confirmed a significant association between a high level of CD62E^+^ microparticles and an increased risk of all major cardiovascular events (Table 2). A high level of CD62E^+^ microparticles was also significantly associated with an increased risk of hospitalization.

**Table 2:** Multivariate regression analysis of the association between higher levels of CD62E^+^ microparticles and outcome.

Outcome	Unadjusted Hazard Ratio (95% CI)	P value	Adjusted Hazard Ratio (95% CI)*	P value*
All major cardiovascular events	2.70 (1.19–6.09)	0.017	3.06 (1.26–7.46)	0.014
Myocardial infarction or new CAD	2.75 (0.73–10.37)	0.135	1.68 (0.35–8.06)	0.517
Revascularization	4.15 (0.46–37.11)	0.203	0.41 (0.02–10.80)	0.596
Hospitalization†	2.91 (1.15–7.38)	0.025	3.33 (1.19–9.36)	0.022
All stroke	1.88 (0.70–5.09)	0.213	2.03 (0.68–6.10)	0.205
Ischemic stroke	2.31 (071–7.50)	0.163	2.28 (0.63–8.25)	0.210
Hemorrhagic stroke	1.02 (0.14–7.28)	0.980	0.94 (0.06–15.17)	0.963
Death from cardiovascular causes	4.14 (0.46–37.02)	0.204	2.22 (0.20–24.80)	0.516
Death from any cause	2.07 (0.52–6.27)	0.304	2.80 (0.63–12.42)	0.174

* Hazard ratios were adjusted for all of the following: age; sex; presence of hypertension, diabetes, hyperlipidemia, smoking status, high cardioembolic risk, or known coronary artery disease; stroke etiology; and concomitant treatment with antiplatelet agents, warfarin, angiotensin-converting enzyme inhibitors, angiotensin receptor blockers, beta blockers, calcium channel blockers, diuretics, or statins.

† Hospitalization was due to cardiovascular events, including recurrent angina, congestive heart failure, myocardial infarction, or stroke. CAD denotes coronary artery disease and CI denotes confidence interval.

### CD31^+^/CD42^−^ and CD31^+^/Annexin-V^+^ microparticles

The number of CD31^+^/Annexin-V^+^ microparticles ranged from 7 to 3684 (mean ± standard error, 213±407), and the number of CD31^+^/CD42^−^ microparticles ranged from 9 to 3902 (232±415). There was not a significant association between these two measures and any of the baseline characteristics or with any of the cardiovascular outcomes (data not shown). The levels of CD62E+ microparticles were not correlated with the levels of CD31^+^/CD42^−^ (Figure S2) or CD31^+^/Annexin-V^+^ microparticles (Figure S3).

## Discussion

Here, we showed that a high level of CD62E^+^ microparticles is associated with an increased risk of major cardiovascular events and hospitalization in patients with old stroke. Because CD62E^+^ microparticles are released from activated endothelial cells [4], our results suggest that endothelial activation participates in the pathogenesis of cardiovascular diseases and is likely to increase the risk of subsequent cardiovascular morbidities. In addition, measurement of CD62E^+^ EMP levels might be helpful for identifying high-risk patients.

The current study is important because it shows a clinical significance of EMPs in patients with stroke history, where few markers of cardiovascular outcome have been identified. Several markers of cardiovascular risk have been identified in normal population or coronary diseases. For example, an increased carotid artery intima-media thickness is a predictor of future vascular events [20], an elevated homocysteine concentration is associated with ischemic heart disease and stroke in healthy populations [21], high C-reactive protein levels are associated with an increased likelihood of cardiovascular events in women [22], and the number of endothelial progenitor cells is predictive of cardiovascular outcome in patients with coronary artery diseases [23]. However, the etiologies of stroke include non-atherosclerotic conditions, such as cardioembolism and hemorrhagic stroke, so the cardiovascular prognosis of stroke patients is expected to differ from that of patients with coronary artery diseases.

Although the patient population in this study was heterogeneous with including all chronic stroke etiologies, it is representative of what is typically encountered in the stroke clinic and therefore clinically relevant. Because the classification of stroke etiologies can be subjective [18], it is more clinically relevant than selecting individual stroke etiologies. Stroke patients with an atherosclerotic etiology often have co-morbidities of cardioembolism and hemorrhagic stroke, further complicating determination of the etiology. In our study, the use of anticoagulation was associated with the higher recurrence of ischemic stroke. This is likely a confounding effect because the patients with high risk factors for cardioembolic stroke, such as atrial fibrillation, were treated with anticoagulation, which could not totally ameliorate the development of subsequent ischemic strokes.

The level of CD62E^+^ microparticles is likely to be an indicator of systemic endothelial pathology rather than of cerebral atherosclerosis itself. This is because higher levels of CD62E^+^ microparticles were associated with the occurrence of all major cardiovascular diseases and of hospitalization, whereas they were not associated with stroke recurrence. However, interpretation and application of these results are limited by the relatively few outcome events that were assessed and because all cardiovascular events and hospitalization comprised most (>20) of the events that occurred. Accordingly, larger studies with more outcome events are needed to confirm the current findings and to extend them in non-stroke populations.

In this study, we enrolled patients who experienced a first-ever stroke at least 3 months prior to enrolment. According to a previous study, newly-generated microparticles are cleared within 10 min from the circulation [24]. Our previous data also showed that the level of EMPs is increased acutely after ischemic stroke and normalized in six days [8]. In the current study, the mean level of CD62E^+^ microparticles in the patients with previous old stroke was 1.75±0.40 (common logarithmic transformed), which is similar to the normal level of CD62E^+^ microparticles in subjects without previous stroke [8]. In addition, there was no correlation between the level of CD62E^+^ microparticles and the time since old stroke onset in the current study. Accordingly, EMPs remain very shortly in the blood, and there must be continuous microparticle production in the high microparticle group. Follow-up measurement of the EMP levels at the end of the study might have provided additional information.

Our results suggest that endothelial activation plays a more important role than apoptosis in the worsening of the cardiovascular condition in patients with stroke history. Unlike the level of CD62E^+^ microparticles, the level of CD31^+^/Annexin-V^+^ apoptotic microparticles was not predictive of cardiovascular outcomes. This contrasts with previous reports that, in patients with coronary artery diseases, the high level of circulating CD31^+^/Annexin-V^+^ microparticles correlates with impairment of coronary endothelial function or future cardiovascular events [25], [26]. The differences between these results and ours are probably due to different endothelial responses underlying stroke and coronary artery disease. When endothelial cells undergo apoptosis, the EMPs released predominantly bear constitutive markers such as CD31 and Annexin-V, whereas activated endothelial cells release EMPs in which inducible markers such as CD62E predominate [4]. CD62E is a member of the selectin family of adhesion molecules expressed primarily on activated vascular endothelium and facilitates the rolling and adhesion of inflammatory cells [4], [27]. Our previous study also showed that patients with extracranial arterial stenosis had higher levels of CD62E^+^ microparticles than those without the stenosis, while CD31^+^/Annexin-V^+^ microparticles was associated with intracranial stenosis [8]. Therefore, the surface marker for detecting EMPs must be chosen according to the characteristics of patient population.

The precise function of CD62E^+^ microparticles remains to be determined. EMPs are likely to be involved in the pathogenesis of cardiovascular diseases by promoting inflammation, vascular dysfunction, and coagulation [5]. EMPs impair endothelium-dependent relaxation by regulating the endothelial nitric oxide transduction pathway [28]–[30], by expressing prothrombotic tissue factor activity [31], and by disseminating proadhesive and procoagulant factors in thrombotic disorders [32]. In addition, EMPs act as vectors for transcellular messages that regulate vascular function [5], and they mediate the activation of endothelium-leukocyte crosstalk by stimulating procoagulant properties of leukocytes [33]. Our current findings suggest that the extent of endothelial cell activation is an indicator of the active pathogenesis of cardiovascular disease. Further improvements in the EMP assay may allow non-invasive, real-time monitoring of vascular endothelial responses and the effects of medicines that prevent vascular damage.

## Supporting Information

Figure S1 **Correlation plots between CD62E^+^ levels and time since stroke onset.** (**A**) The two variables were not correlated each other (Pearson's correlation coefficient = 0.057, P = 0.327). CD62E^+^ levels are counts/µL of platelet-poor plasma. (**B**) Even after common logarithmic transformation, the level of CD62E microparticles was not correlated with the time since stroke onset (Pearson's correlation coefficient = 0.065, P = 0.262).
(TIF)

Figure S2 **Correlation plots between CD62E^+^ and CD31^+^/CD42^−^ microparticles.** The two measurements were not correlated each other (Pearson's correlation coefficient = 0.040, P = 0.487). Values are counts/µL of platelet-poor plasma.
(TIF)

Figure S3 **Correlation plots between CD62E^+^ and CD31^+^/Annexin-V^−^ microparticles.** The two measurements were not correlated each other (Pearson's correlation coefficient = 0.028, P = 0.636). Values are counts/µL of platelet-poor plasma.
(TIF)

Table S1 **Baseline characteristics of the patients.**
(DOCX)
