# Supplementary figures and images for: Circulating CD62E+ Microparticles and Cardiovascular Outcomes
Source: PLoS One. 2012 Apr 26;7(4):e35713. doi: 10.1371/journal.pone.0035713 (PMC3338519; doi:10.1371/journal.pone.0035713)

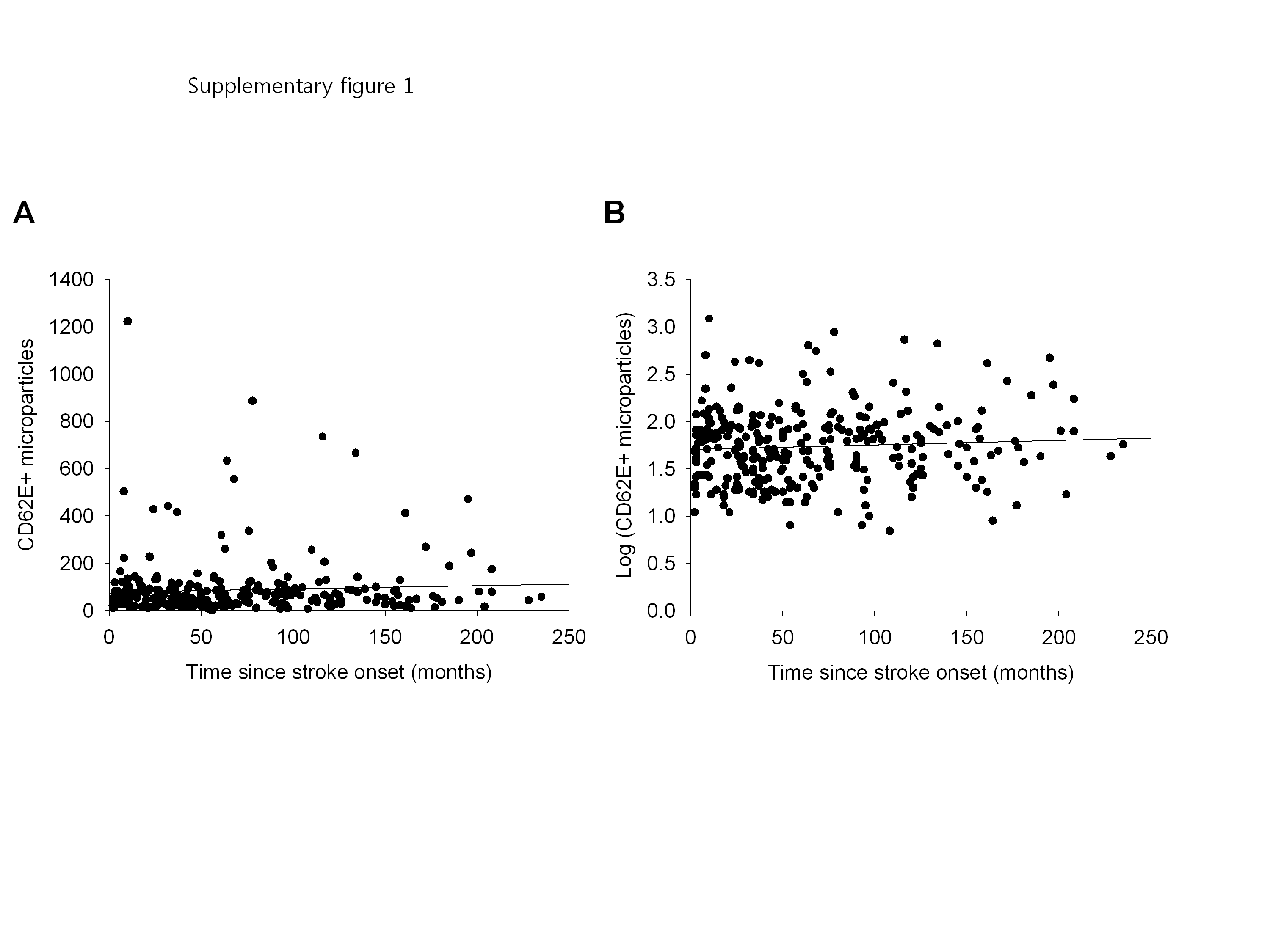

Supplement: Figure S1 — Correlation plots between CD62E+ levels and time since stroke onset. (A) The two variables were not correlated each other (Pearson's correlation coefficient = 0.057, P = 0.327). CD62E+ levels are counts/µL of platelet-poor plasma. (B) Even after common logarithmic transformation, the level of CD62E microparticles was not correlated with the time since stroke onset (Pearson's correlation coefficient = 0.065, P = 0.262). (TIF) [file pone.0035713.s001.tif]

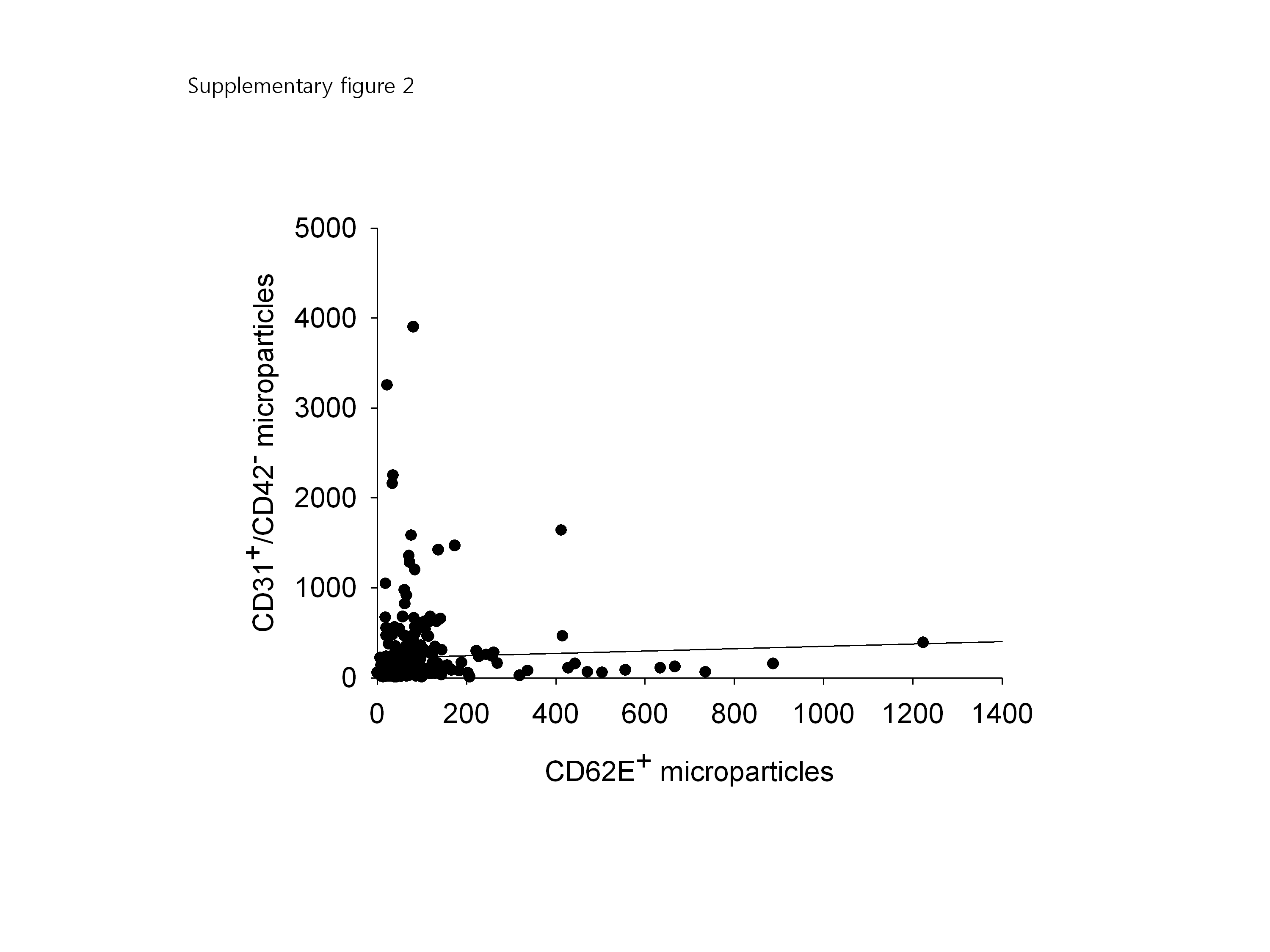

Supplement: Figure S2 — Correlation plots between CD62E+ and CD31+/CD42− microparticles. The two measurements were not correlated each other (Pearson's correlation coefficient = 0.040, P = 0.487). Values are counts/µL of platelet-poor plasma. (TIF) [file pone.0035713.s002.tif]

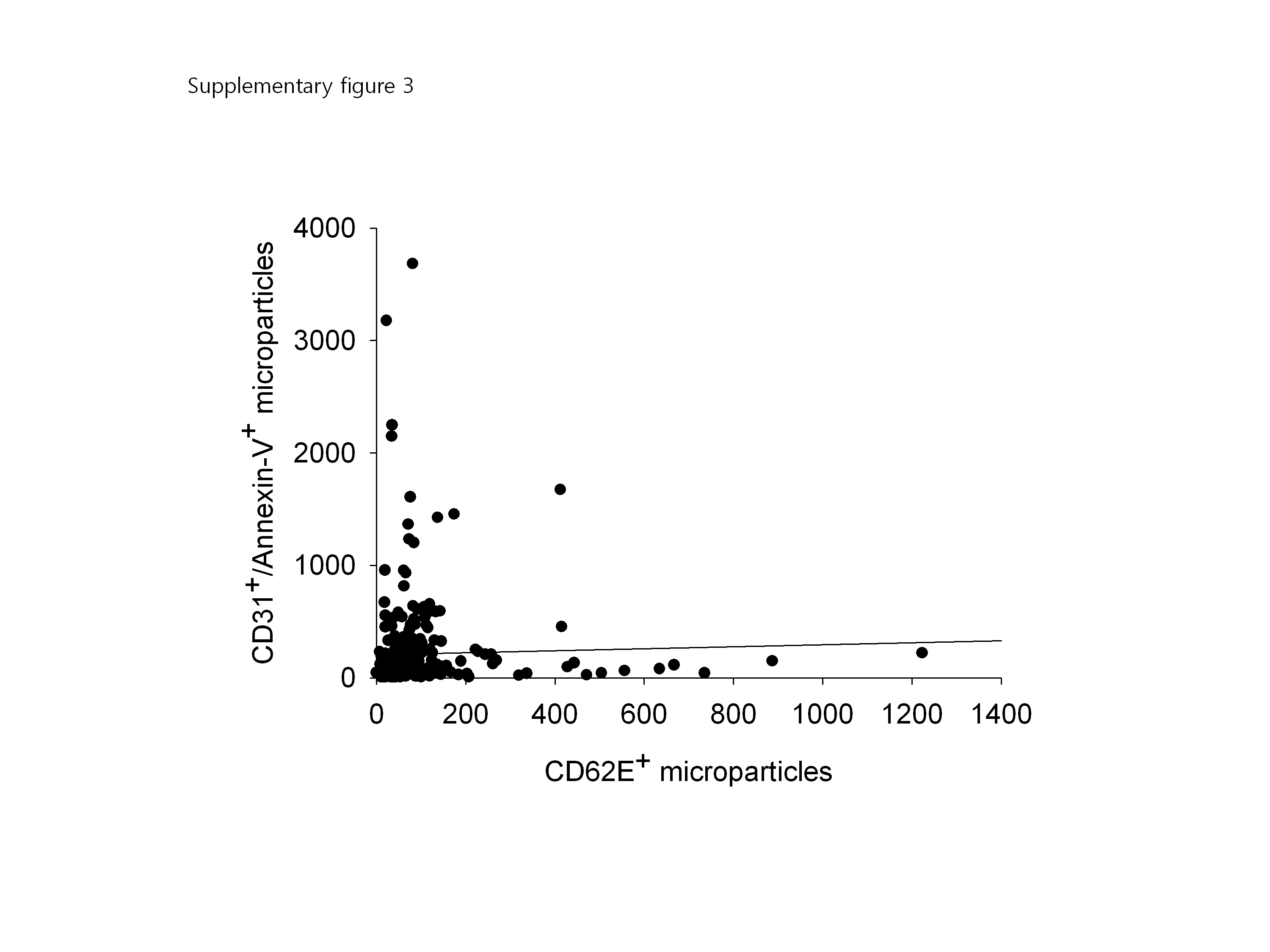

Supplement: Figure S3 — Correlation plots between CD62E+ and CD31+/Annexin-V− microparticles. The two measurements were not correlated each other (Pearson's correlation coefficient = 0.028, P = 0.636). Values are counts/µL of platelet-poor plasma. (TIF) [file pone.0035713.s003.tif]
