# Supplementary material for: Circulating CD62E+ Microparticles and Cardiovascular Outcomes
Source: PLoS One. 2012 Apr 26;7(4):e35713. doi: 10.1371/journal.pone.0035713 (PMC3338519; doi:10.1371/journal.pone.0035713)
Supplement: Table S1 — Baseline characteristics of the patients. (DOCX) [file pone.0035713.s004.docx]

**Table S1.** Baseline characteristics of the patients.

| Characteristics | Total (N=298) | Low CD62E+ microparticle group (N=149) | High CD62E+ microparticle group (N=149) | P value* |
| --- | --- | --- | --- | --- |
| Age – y (mean ± SD) | 64.7±9.9 | 64.4±10.4 | 65.1±9.4 | 0.528 |
| Male gender – *n* (%) | 102 (34.2) | 55 (36.9) | 47 (31.5) | 0.329 |
| Medical history – *n* (%) |  |  |  |  |
| Hypertension | 246 (82.6) | 119 (79.9) | 127 (85.2) | 0.222 |
| Diabetes mellitus | 81 (27.2) | 35 (23.5) | 46 (30.9) | 0.152 |
| Hyperlipidemia | 58 (19.5) | 29 (19.5) | 29 (19.5) | 1.000 |
| Current Smoking | 27 (9.1) | 13 (8.7) | 14 (9.4) | 0.840 |
| Family history of CAD | 100 (33.6) | 45 (30.2) | 55 (36.9) | 0.220 |
| CE high risk | 41 (13.8) | 21 (14.1) | 20 (13.4) | 0.866 |
| Known CAD | 25 (8.4) | 9 (6.0) | 16 (10.7) | 0.144 |
| Months since stroke onset | 65.4±52.6 | 63.8±50.4 | 67.0±54.8 | 0.598 |
| Body mass index | 24.1±3.0 | 23.9±2.9 | 24.3±3.0 | 0.340 |
| Framingham risk score | 7.3±3.0 | 7.4±3.3 | 7.2±2.9 | 0.588 |
| Medication – *n* (%) |  |  |  |  |
| Antiplatelet agents -single | 191 (64.1) | 101 (67.8) | 90 (60.4) | 0.411 |
| Antiplatelet agents -dual | 55 (18.5) | 25 (16.8) | 30 (20.1) |  |
| Anticoagulation | 39 (13.1) | 20 (13.4) | 19 (12.8) | 0.864 |
| ACE inhibitors / ATR-blockers | 149 (50.0) | 74 (49.7) | 75 (50.0) | 0.908 |
| Beta-blockers | 59 (19.8) | 31 (20.8) | 28 (18.8) | 0.663 |
| Calcium-channel blockers | 113 (37.9) | 54 (36.2) | 59 (39.6) | 0.551 |
| Diuretics | 64 (21.5) | 34 (22.8) | 30 (20.1) | 0.573 |
| Statins | 75 (25.2) | 35 (23.5) | 40 (26.8) | 0.445 |
| Stroke etiology group – *n* (%) |  |  |  |  |
| Atherothrombotic | 85 (28.5) | 47 (31.5) | 38 (25.5) | 0.248 |
| Lacunar stroke | 123 (41.3) | 64 (43.0) | 59 (39.6) | 0.556 |
| Undetermined | 11 (3.7) | 4 (2.7) | 7 (4.7) | 0.357 |
| Transient ischemic attack | 19 (6.4) | 7 (4.7) | 12 (8.1) | 0.236 |
| Cardioembolic | 34 (11.4) | 18 (12.1) | 16 (10.7) | 0.716 |
| Intracerebral hemorrhage | 26 (8.7) | 9 (6.0) | 17 (11.4) | 0.101 |

**P* values were determined by Student’s *t*-test for continuous values with a normal distribution, by Mann-Whitney *U* test for continuous variables with a non-normal distribution, or by chi-square test for categorical values. ACE denotes angiotensin converting enzyme; ATR, angiotensin receptor; CAD, coronary artery disease; CE, cardioembolic; and SD, standard deviation.
